# Causal associations between platelet count, alcohol consumption, and the risk of liver hepatocellular carcinoma based on a Mendelian randomization study

**DOI:** 10.3389/fendo.2024.1400573

**Published:** 2024-05-22

**Authors:** Lihua Yu, Leisheng Wang, Yuzheng Xue, Yilin Ren, Tianhao Liu, Hao Hu

**Affiliations:** ^1^ Department of Gastroenterology, Affiliated Hospital of Jiangnan University, Wuxi, China; ^2^ Hepatobiliary and Pancreatic Surgery, Affiliated Hospital of Jiangnan University, Wuxi, China; ^3^ School of Medicine, Jiangnan University, Wuxi, China; ^4^ Wuxi Institute of Hepatobiliary Surgery, Wuxi, China

**Keywords:** platelet count, alcohol consumption, Alzheimer’s disease, liver hepatocellular carcinoma, Mendelian randomization

## Abstract

**Background and aims:**

Liver hepatocellular carcinoma (LIHC) exhibits a multifactorial etiology, insidious onset, and a significantly low 5-year survival rate. We aimed to evaluate the causal impact of exposure factors (Alzheimer’s disease, platelet count, ambidextrousness, cigarettes smoked per day, alcohol consumption, and endocarditis) on the risk of LIHC using a two-sample Mendelian randomization (MR) study.

**Methods:**

Independent single nucleotide polymorphisms (SNPs) strongly associated with Alzheimer’s disease, platelet count, ambidextrousness, daily cigarette consumption, alcohol intake, and endocarditis were selected as instrumental variables (IVs) from the corresponding genome-wide association studies (GWAS). Genetic summary statistics for LIHC came from a GWAS that included 168 cases and 372,016 controls of European individuals. Multivariable MR analyses were performed to find the causal association between 6 exposure factors and LIHC risk. The inverse-variance weighted (IVW)-MR was employed as the primary analysis, and the MR-Egger regression, LASSO regression, and weighted Median approaches were performed as complementary analyses.

**Results:**

Multivariable MR analysis showed causal association between Alzheimer’s disease [Odds ratio (OR) = 0.9999, 95% confidence intervals (CI) = 0.9998-0.9999, p = 0.0010], platelet count (OR = 0.9997, 95% CI = 0.9995-0.9999, p = 0.0066), alcohol consumption (OR = 0.9994, 95% CI = 0.9990-0.9999, p = 0.0098) and the LIHC outcome. After IVW-MR, MR-Egger and LASSO tests, the results are still significant. Next, we used different MR Methods to analyze platelet count, alcohol consumption, and Alzheimer’s disease separately. Moreover, both funnel plots and MR-Egger intercepts provided compelling evidence to refute the presence of directional pleiotropy in the association between platelet count, alcohol consumption, Alzheimer’s disease and the risk of LIHC. The IVW-MR analysis revealed a significant causal association between an elevated platelet count and a reduced risk of LIHC (OR = 0.9996, 95% CI= 0.9995-0.9998, p = 0.0005). Similarly, the analysis of weighted median revealed a negative correlation between platelet count and the risk of LIHC (OR = 0.9995, 95% CI = 0.9993-0.9999; p = 0.0160). Conversely, we observed a positive causal effect of alcohol consumption on the incidence of LIHC (OR = 1.0004, 95% CI = 0.9999-1.0009). However, no significant causal relationship was found between alcohol assumption, Alzheimer’s disease, and LIHC susceptibility.

**Conclusions:**

A significant causal relationship exists between platelet count, alcohol consumption, Alzheimer’s disease, and an increased risk of LIHC. The study presents compelling evidence for a genetically predicted decreased susceptibility to LIHC based on platelet count. The research implies that elevated platelet count may serve as a protective mechanism against LIHC. These findings may inform clinical strategies for LIHC prevention

## 1 Introduction

Liver hepatocellular carcinoma (LIHC) is the most common primary liver cancer and ranks as the third leading cause of cancer-related deaths globally (1). LIHC typically occurs in individuals with chronic liver conditions, primarily resulting from viral hepatitis, alcohol-induced liver disease, or non-alcoholic fatty liver disease (2). Currently, there is a lack of established screening programs for early detection due to the difficulty in identifying symptoms during the initial stages of the disease (3). While understanding symptom profiles associated with LIHC may offer some potential for early diagnosis, it remains crucial to identify clinical and biochemical factors that can assist in identifying high-risk subpopulations for timely intervention through imaging or participation in screening studies (4). Enhancing prevention strategies and developing innovative therapies are essential to improve outcomes for patients with LIHC.

Platelets are increasingly being recognized for their role in inflammation and the progression of cancer, as they release various substances that contribute to tumor development (5). The use of anti-platelet medications such as aspirin holds promise in the treatment of cancer, as indicated by a systematic review and meta-analysis showing a decreased risk of liver cancer occurrence and improved survival rates related to liver health (6). In an external validation group comprising 525 patients with cirrhosis and liver cancer, individuals with low platelet count and high mean platelet volume demonstrated significantly prolonged overall survival (OS) based on both univariate and multivariate analysis (7); however, it is important to note that this study has a retrospective design with exploratory nature. Therefore, further confirmation through prospective randomized controlled trials is necessary to validate these findings.

Globally, cancer and dementia are prominent causes of mortality that tend to escalate with advancing age (8). Numerous epidemiological investigations have indicated a negative correlation between these two ailments, specifically in relation to Alzheimer’s dementia (9, 10). No existing study has demonstrated a definitive cause-and-effect link between Alzheimer’s dementia and LIHC. Furthermore, certain research findings have linked tobacco smoking (11–13), alcohol consumption (14), as well as streptococcus infection (15) to heightened susceptibility for fatality due to chronic liver disease; however, there is a scarcity of comprehensive analyses regarding their temporal patterns. Furthermore, it is important to mention that the majority of previous studies examining their correlation were primarily based on observation or cross-sectional analysis. This factor may have introduced potential confounding variables and thus produced inconclusive results. As a result, it is crucial to adopt a fresh investigative approach in order to uncover the precise impact of these 6 exposure factors on the risk of LIHC.

The Mendelian randomization (MR) approach was employed to establish causal links between exposures and outcomes by utilizing single nucleotide polymorphisms (SNPs) as instrumental variables (IVs) (20). SNPs were randomly assigned from parents to offspring during conception, ensuring that the MR method remained unaffected by confounding or reverse causation, similar to the random assignment in randomized controlled trials (20). In this investigation, we conducted a two-sample MR analysis to explore the connection between exposure factors (Alzheimer’s disease, platelet count, ambidextrousness, daily cigarette consumption, alcohol intake, and endocarditis) and the risk of LIHC.

## 2 Materials and methods

### 2.1 Study design

This MR investigation utilizes summary-level data from publicly accessible genome-wide association studies (GWAS). All of these studies have obtained approval from the appropriate institutional review boards, and participants have provided informed consent.

### 2.2 Exposure data sources

In this study, we selected six variables for investigation: Alzheimer’s disease (dataset: ebi-a-GCST002245), platelet count (dataset: ebi-a-GCST004603), ambidextrousness (dataset: ebi-a-GCST90013420), daily cigarette consumption (dataset: ieu-b-4826), alcohol consumption (dataset: ieu-b-4834), and endocarditis (dataset: ieu-b-4972). The exposure data for late-onset Alzheimer’s disease were obtained through genome-wide association studies (GWAS), which involved genotyping approximately 7,022,150 single nucleotide polymorphisms (SNPs). This study included a cohort of individuals with European ancestry comprising 17,008 cases and 37,154 control adults. The platelet count exposure data was derived from a GWAS analysis utilizing approximately 29,148,896 SNPs. The ambidextrousness exposure data was obtained through a GWAS using 11,683,993 SNPs in a cohort consisting of 47,637 cases and 1,422,823 controls. The data on daily cigarette consumption was obtained through a GWAS using 7,227,329 SNPs in a cohort consisting of both males and females, totaling 24,784 individuals. The data on alcohol consumption was obtained through a GWAS using 7,914,362 SNPs in a cohort comprising both males and females, totaling 83,626 individuals. The data on endocarditis exposure was obtained through a GWAS using 12,243,455 SNPs in a cohort consisting of 1,080 cases and 485,404 controls. The study population was limited to individuals of European descent.

### 2.3 Outcome sources

The UK Biobank is a prospective study that enrolled half a million volunteers aged 37 to 73 from various regions of the United Kingdom between 2006 and 2010. Detailed information regarding the involvement of patients and the public can be accessed online. All participants in this study provided written consent after being fully informed, and it received approval from the National Research Ethics Services Board, North-West-Haydock. The genetic summary statistics for LIHC were obtained from a GWAS involving individuals of European descent, consisting of 168 cases and 372,016 controls (ieu-b-4953). All research procedures strictly adhere to the ethical principles outlined in the Helsinki Declaration for Medical Research established by the World Medical Association.

### 2.4 Selection of instrumental variables

We selected IVs linked to LIHC at the genome-wide significance levels from GWAS with p < 5.0×10-8. To ensure independence among the IVs, we utilized the “TwoSampleMR” package to set a linkage disequilibrium (LD) threshold of R2 < 0.001 for the 1000 Genomes European data and an aggregation distance of 10,000 kb. After extracting relevant information on each SNP’s effect allele, including β value, standard error, and P-value, we calculated the variance explanation ratio (R2) and F statistic to quantify tool strength as follows: R2 = 2 × MAF × (1 - MAF) × β^2 and F = R2(n - k - 1)/(k(1 - R2)), where MAF represents the minor allele frequency of SNPs used as IVs, n denotes sample size, and k signifies the number of IVs employed. The choice of statistical methods and instrumental variables in our study was guided by their ability to provide robust and reliable causal inferences. Specifically, we selected MR methods such as IVW and MR-Egger regression for their strengths in addressing different aspects of potential biases, and SNPs were chosen as instrumental variables based on stringent criteria to ensure their relevance and independence in relation to the exposure and outcome.

### 2.5 Statistical analyses

#### MR analysis

The primary analysis selected was the inverse-variance-weighted Mendelian randomization (IVW-MR) method (16), while supplementary tools such as MR-Egger regression, weighted median (17), simple mode, and weighted mode methods were also utilized. Despite the inherent advantages of Mendelian randomization in mitigating confounding biases, we acknowledge the potential for residual confounding factors that could influence our observations. We have attempted to minimize this impact by using robust statistical methods and sensitivity analyses, yet the possibility of unmeasured confounders remains a limitation of our study. Initially, the IVW method utilizes the Wald estimator and Delta method to calculate rate estimates for individual SNPs, which are then combined to derive the primary causal estimate. When heterogeneity was statistically significant, the random effect model was utilized. Otherwise, the fixed effect model was utilized. The MR-Egger regression technique was utilized to evaluate potential levels of pleiotropy, with a significance level of P < 0.05 indicating the presence of potential pleiotropy at the SNP level (18). The weighted median MR approach introduces a new methodology that provides a consistent estimator even in the presence of significant heterogeneity. This estimator effectively manages type I errors, improving the ability to identify causal effects and ensuring stability even when more than 50% of information is derived from invalid instrumental variables (17). The MR-Egger method can identify and correct potential pleiotropy and provide a relatively consistent estimate (18). For multivariable MR-IVW analyses, multivariable MR-IVW was performed as the primary analysis. The least absolute shrinkage and selection operator (LASSO) regression provides the best estimation for moderate-to-high levels of pleiotropy and valid inference (19).

#### Sensitivity analysis

The heterogeneity among SNPs was assessed using Cochran’s Q statistic (20). The MR-Egger intercept method was used to test whether genetic variants have pleiotropic effects on infections. To identify potential outlier SNPs, leave-one-out methods were applied (21). The effect size of individual SNPs on the risk of LIHC associated with exposure factors was visualized using a forest plot. Causal effects of exposure on LIHC were evaluated through scatter plots. Additionally, the symmetrical distribution of selected SNPs was demonstrated using a funnel plot. Statistical significance for sensitivity analysis was defined as p<0.05. R software version 4.1.0 was used for all data analyses.

## 3 Results

### 3.1 Multivariable MR analyses

**Figure 1:**
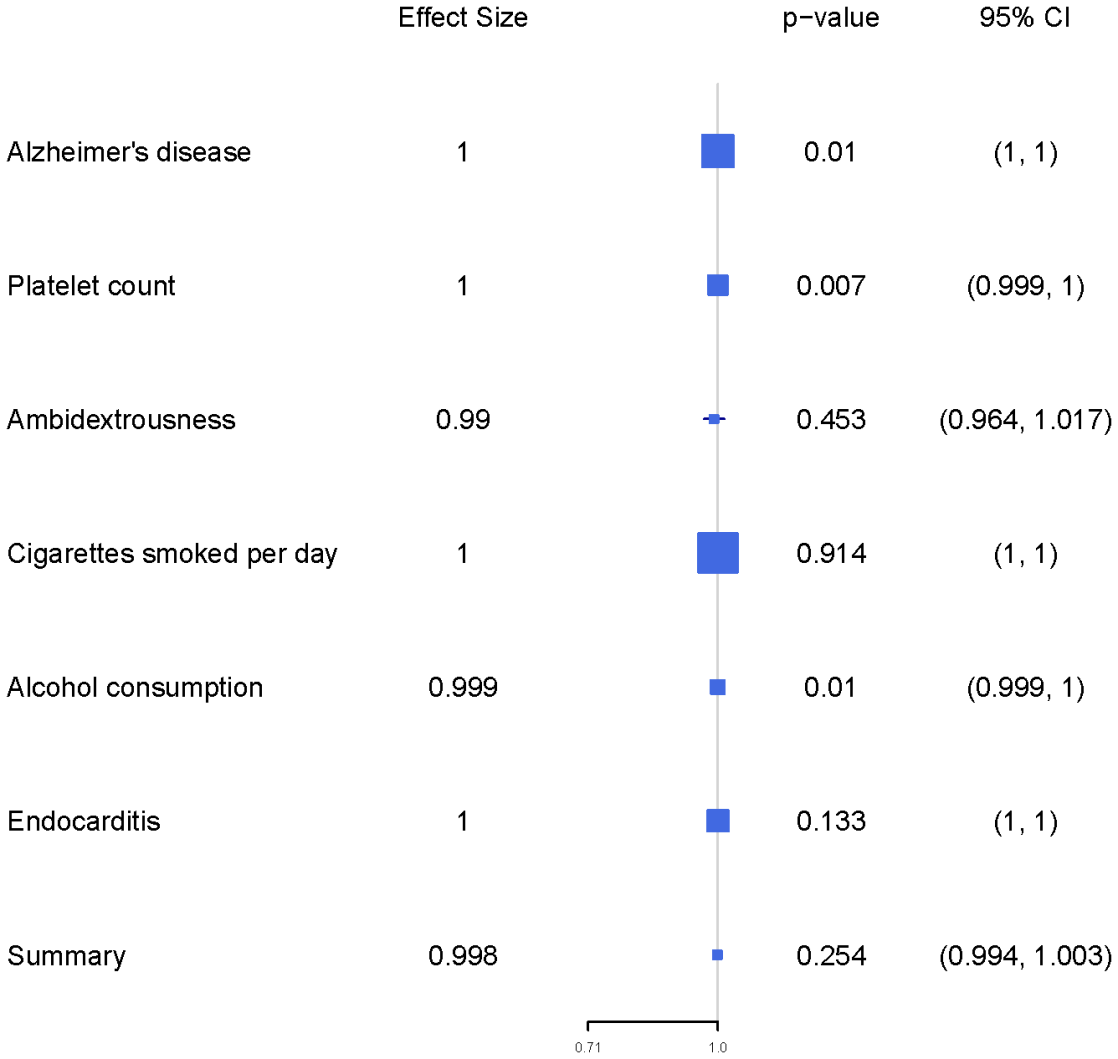
The forest plot presents the findings of a multivariable Mendelian Randomization (MR) analysis.

We estimated mutually the effects of 6 exposure factors, including Alzheimer’s disease, platelet count, ambidextrousness, daily cigarette consumption, alcohol consumption, and endocarditis on LIHC using multivariable MR analyses. We observed directly inverse effect of Alzheimer’s disease (OR = 0.9999, 95% CI = 0.9998-0.9999, p = 0.0100), platelet count (OR = 0.9995, 95% CI = 0.9995-0.9999, p = 0.0066), and alcohol consumption (OR = 0.9990, 95% CI = 0.9997-0.9999, p = 0.0098) on LIHC (**Figure 1**; **Table 1**).

**Table 1:** The results of multivariable Mendelian Randomization analyses.

No.	id.exposure	exposure	id.outcome	outcome	nsnp	b	se	pval	lo_ci	up_ci	or	or_lci95	or_uci95
1	ebi-a-GCST002245	Alzheimer's disease (late onset)	ieu-b-4953	LIHC	8	-0.00013719	5.33E-05	0.01000504	-0.0002	######	0.99986	0.99976	0.99997
2	ebi-a-GCST004603	Platelet count	ieu-b-4953	LIHC	172	-0.00030326	0.00011171	0.00663247	-0.0005	######	0.9997	0.99948	0.99992
3	ebi-a-GCST90013420	Ambidextrousness	ieu-b-4953	LIHC	0	-0.01017807	0.01355612	0.45276695	-0.0367	0.01639	0.98987	0.96392	1.01653
4	ieu-b-4826	Cigarettes smoked per day	ieu-b-4953	LIHC	3	-4.39E-06	4.06E-05	0.91382739	######	7.51E-05	1	0.99992	1.00008
5	ieu-b-4834	Alcohol consumption	ieu-b-4953	LIHC	0	-0.00057839	0.00022403	0.00983149	-0.001	-0.0001	0.99942	0.99898	0.99986
6	ieu-b-4972	Endocarditis	ieu-b-4953	LIHC	0	-0.00014359	9.55E-05	0.13270298	-0.0003	4.36E-05	0.99986	0.99967	1.00004

**Figure 2:**
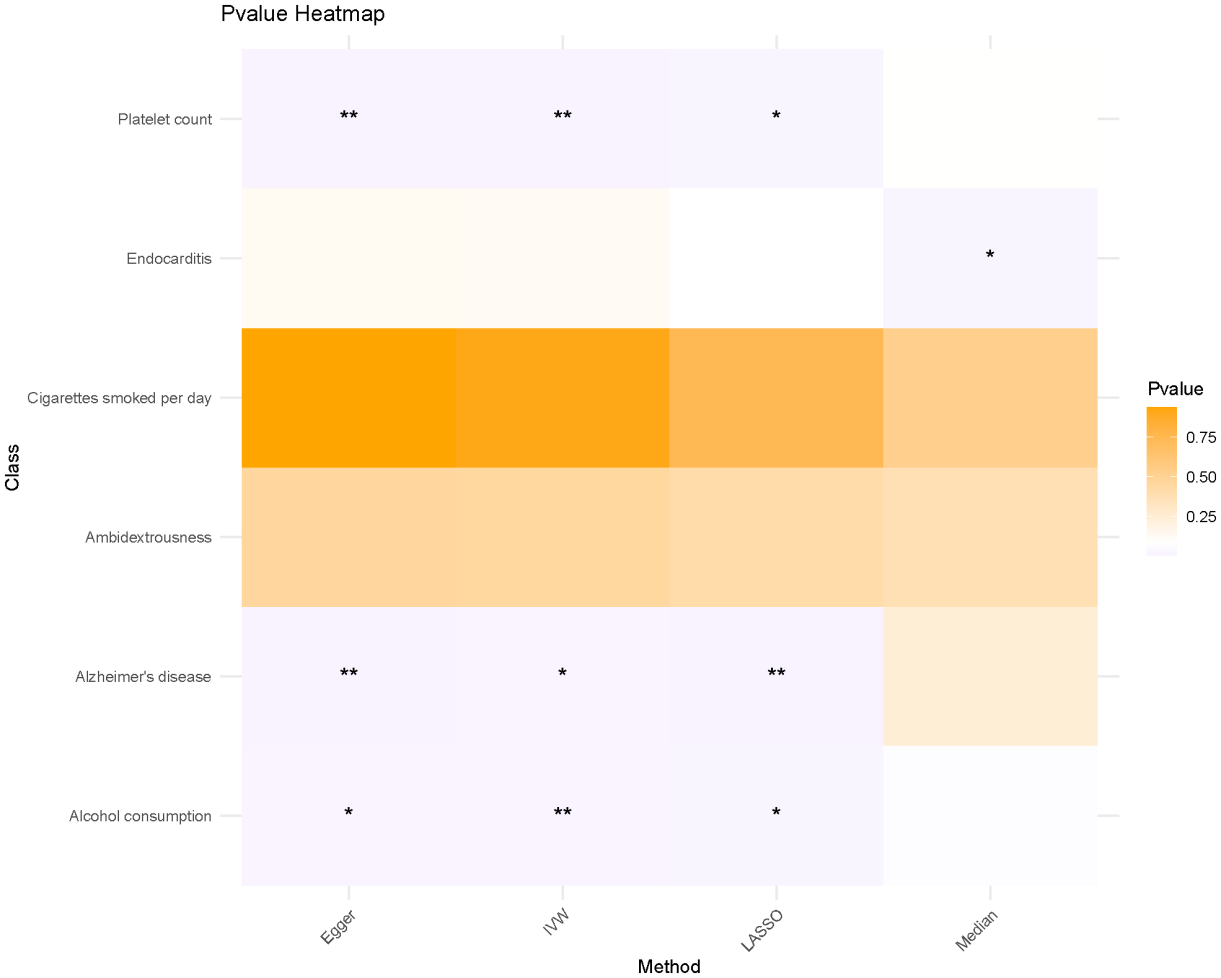
The heat map illustrates the impact of various MR methods on each exposure factor. * means p<0.05, ** means P<0.01.

Using four distinct MR methods, we identified significant causal associations between Alzheimer’s disease, platelet count, and alcohol consumption with the risk of LIHC in the IVW analysis. Similar conclusions were drawn from Egger analysis and LASSO analysis, while only a significant causal relationship between endocarditis and LIHC risk was observed using the Median method. Notably, LASSO-MR and IVW analyses yielded the most robust causal evidence for Alzheimer’s disease, platelet count, and alcohol intake respectively (**Figure 2**; **Table 2**). To address the apparent contradiction in our findings regarding the relationship between alcohol consumption, Alzheimer’s disease, and LIHC susceptibility, we conducted further sensitivity analyses. These analyses suggest that the initial non-significant association may be attributed to the limited power of our dataset to detect weaker causal relationships in the context of multiple testing and complex interactions among the studied variables.

**Table 2:** The effect of different MR Methods on each exposure factor.

Method	Exposure	Estimate	StdError	CILower	CIUpper	Pvalue
IVW	exposure_1	-0.0001372	5.33E-05	-0.000242	-3.28E-05	0.01001
IVW	exposure_2	-0.0003033	0.0001117	-0.000522	-8.43E-05	0.00663
IVW	exposure_3	-0.0101781	0.0135561	-0.036748	0.01639143	0.45277
IVW	exposure_4	-4.39E-06	4.06E-05	-8.39E-05	7.51E-05	0.91383
IVW	exposure_5	-0.0005784	0.000224	-0.001017	-0.0001393	0.00983
IVW	exposure_6	-0.0001436	9.55E-05	-0.000331	4.36E-05	0.1327
Egger	exposure_1	-0.0001468	5.61E-05	-0.000257	-3.68E-05	0.0089
Egger	exposure_2	-0.0002981	0.0001123	-0.000518	-7.80E-05	0.00794
Egger	exposure_3	-0.0099459	0.0135892	-0.03658	0.01668839	0.46423
Egger	exposure_4	-3.03E-06	4.07E-05	-8.28E-05	7.68E-05	0.94067
Egger	exposure_5	-0.0005774	0.0002245	-0.001017	-0.0001375	0.0101
Egger	exposure_6	-0.0001428	9.57E-05	-0.00033	4.48E-05	0.13573
LASSO	exposure_1	-0.0001422	5.09E-05	-0.000242	-4.24E-05	0.00524
LASSO	exposure_2	-0.0002545	0.0001071	-0.000464	-4.46E-05	0.0175
LASSO	exposure_3	-0.0105548	0.0129459	-0.035928	0.01481859	0.4149
LASSO	exposure_4	1.28E-05	3.89E-05	-6.36E-05	8.91E-05	0.7433
LASSO	exposure_5	-0.0005061	0.000215	-0.000927	-8.48E-05	0.01856
LASSO	exposure_6	-0.0001595	9.20E-05	-0.00034	2.08E-05	0.08294
Median	exposure_1	-0.0001052	8.98E-05	-0.000281	7.09E-05	0.24167
Median	exposure_2	-0.000266	0.0001567	-0.000573	4.11E-05	0.08959
Median	exposure_3	-0.0130053	0.0147294	-0.041874	0.01586383	0.37727
Median	exposure_4	-3.27E-05	5.18E-05	-0.000134	6.90E-05	0.52873
Median	exposure_5	-0.000457	0.0002477	-0.000943	2.85E-05	0.06503
Median	exposure_6	-0.0002484	0.0001039	-0.000452	-4.48E-05	0.0168

### 3.2 Causal association between elevated platelet count and decreased risk of LIHC

**Figure 3:**
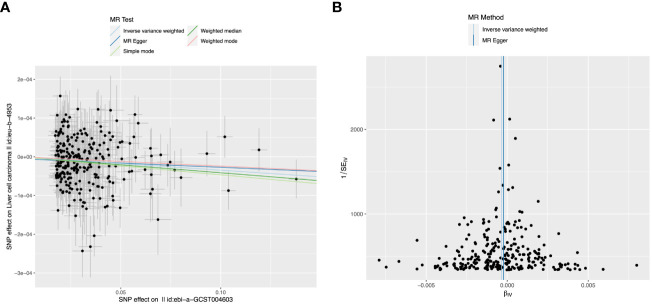
The causal relationship between platelet count and liver hepatocellular carcinoma (LIHC) risk. **(A)** The scatter plot visually represents the causal effect of platelet count on LIHC risk, with the slope of the line indicating the strength of the causal association. **(B)** The forest plot visually depicts the causal impact of individual single nucleotide polymorphisms (SNPs) of platelet count on susceptibility to LIHC. The funnel plots are used to visualize the overall heterogeneity of MR estimates for the effect of platelet count on the risk of LIHC. A leave-one-out plot is used to visualize the causal effect of platelet count on the risk of LIHC when one SNP is excluded.

Considering that multivariate MR Analyses showed significant causal relationships between platelet count, alcohol intake, and Alzheimer’s disease and LIHC risk, we then used different MR Analyses to verify the relationship between these three exposure factors and LIHC susceptibility. Detailed information about the SNPs of LIHC on platelet count is shown in **Supplementary Table 1**. There is a causal relationship between reduced platelet count and increased risk of LIHC using the IVW-MR method (OR = 0.9997, 95% CI = 0.9995-0.9998, p=0.0005), and weighted median method (OR = 0.9996, 95% CI = 0.9993-0.9999, p = 0.0160) (**Table 3**). No heterogeneity (MR Egger, p=0.2196; IVW, p=0.2261) and no potential pleiotropy (MR-Egger, intercept= -5.05E-06, p=0.4927) were observed in the MR analysis. The scatter plot demonstrated the negative causal association between platelet count and the risk of LIHC (**Figure 3A**). The forest plot displays the effect size for every single SNP on the risk of LIHC and shows that causality existed between platelet count and the occurrence of LIHC (**Figure 3B**). The funnel plot showed the selected SNPs were distributed symmetrically. The leave-1-out suggested that no SNPs had an important impact on the estimated causal association.

**Table 3:** Causal effect of platelet count on LIHC risk using multiple MR analyses.

id.exposure	outcome	method	nsnp	b	se	pval	lo_ci	up_ci	or	or_lci95	or_uci95
ebi-a-GCST004603	Liver cell carcinoma || id:ieu-b-4953	MR Egger	272	-0.0002	0.0002	0.2879	-0.0006	0.0002	0.9998	0.9994	1.0002
ebi-a-GCST004603	Liver cell carcinoma || id:ieu-b-4953	Weighted median	272	-0.0004	0.0002	0.016	-0.0007	-0.0001	0.9996	0.9993	0.9999
ebi-a-GCST004603	Liver cell carcinoma || id:ieu-b-4953	Inverse variance weighted	272	-0.0003	0.0001	0.0005	-0.0005	-0.0002	0.9997	0.9995	0.9998
ebi-a-GCST004603	Liver cell carcinoma || id:ieu-b-4953	Simple mode	272	-0.0005	0.0004	0.2544	-0.0013	0.0003	0.9995	0.9987	1.0003
ebi-a-GCST004603	Liver cell carcinoma || id:ieu-b-4953	Weighted mode	272	-0.0002	0.0002	0.2642	-0.0007	0.0002	0.9998	0.9993	1.0002

### 3.3 MR analysis between causal association between alcohol assumption, Alzheimer’s disease, and LIHC risk

**Figure 4:**
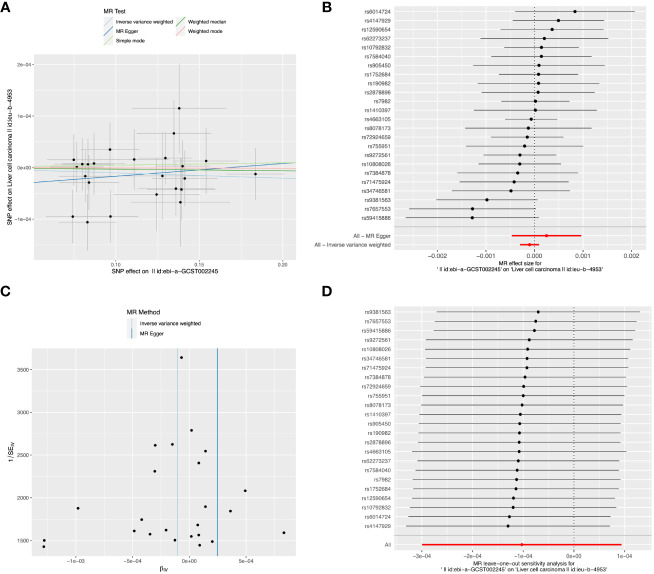
The causal relationship between Alzheimer’s disease and LIHC risk. **(A)** The scatter plot visually represents the causal effect of Alzheimer’s disease on LIHC risk, with the slope of the line indicating the strength of the causal association. **(B)** The forest plot visually depicts the causal impact of individual SNPs of Alzheimer’s disease on susceptibility to LIHC. **(C)** The funnel plots are used to visualize the overall heterogeneity of MR estimates for the effect of Alzheimer’s disease on the risk of LIHC. **(D)** A leave-one-out plot is used to visualize the causal effect of Alzheimer’s disease on the risk of LIHC when one SNP is excluded.

**Figure 5:**
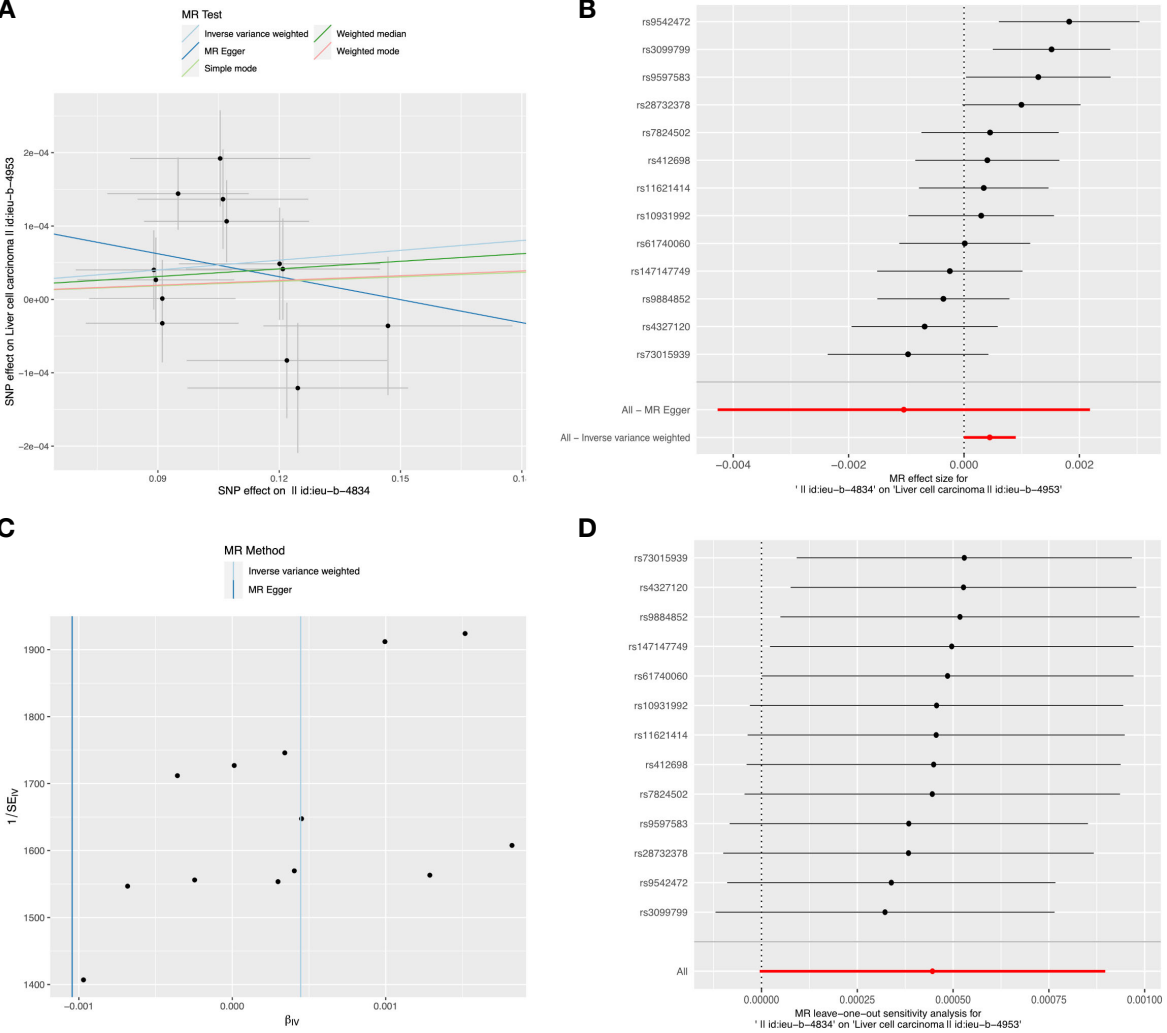
The causal relationship between alcohol assumption and LIHC risk. **(A)** The scatter plot visually represents the causal effect of alcohol assumption on LIHC risk, with the slope of the line indicating the strength of the causal association. **(B)** The forest plot visually depicts the causal impact of individual SNPs of alcohol assumption on susceptibility to LIHC. **(C)** The funnel plots are used to visualize the overall heterogeneity of MR estimates for the effect of alcohol assumption on the risk of LIHC. **(D)** A leave-one-out plot is used to visualize the causal effect of alcohol assumption on the risk of LIHC when one SNP is excluded.

Detailed information about SNPs of LIHC on alcohol assumption and Alzheimer’s disease (**Supplementary Tables 2**, **3**). The primary IVW-MR method showed no causality between alcohol assumption (**Table 4**), Alzheimer’s disease, and LIHC risk. The other MR method results were consistent with IVWs (**Table 5**). The scatter plots, the forest plots, the funnel plots, and the leave-one-out plots of LIHC risk for alcohol assumption and Alzheimer’s disease are displayed in **Figures 4**, **5**, respectively.

**Table 4:** Causal effect of Alzheimer’s disease on LIHC risk using multiple MR analyses.

id.exposure	outcome	method	nsnp	b	se	pval	lo_ci	up_ci	or	or_lci95	or_uci95
ebi-a-GCST002245	Liver cell carcinoma || id:ieu-b-4953	MR Egger	24	0.00024701	0.000364733	0.505316629	-0.000467866	0.000961886	1.00024704	0.999532243	1.000962348
ebi-a-GCST002245	Liver cell carcinoma || id:ieu-b-4953	Weighted median	24	-3.09E-05	0.000136677	0.820966198	-0.000298817	0.000236956	0.99996907	0.999701228	1.000236984
ebi-a-GCST002245	Liver cell carcinoma || id:ieu-b-4953	Inverse variance weighted	24	-0.000102716	0.000100488	0.306701631	-0.000299673	9.42E-05	0.999897289	0.999700372	1.000094246
ebi-a-GCST002245	Liver cell carcinoma || id:ieu-b-4953	Simple mode	24	4.64E-05	0.000225734	0.838911365	-0.000396027	0.000488849	1.000046412	0.999604051	1.000488969
ebi-a-GCST002245	Liver cell carcinoma || id:ieu-b-4953	Weighted mode	24	-6.05E-06	0.00017815	0.973208272	-0.000355222	0.000343125	0.999993951	0.999644841	1.000343184

**Table 5:** Causal effect of alcohol assumption on LIHC risk using multiple MR analyses.

id.exposure	outcome	method	nsnp	b	se	pval	lo_ci	up_ci	or	or_lci95	or_uci95
ieu-b-4834	Liver cell carcinoma || id:ieu-b-4953	MR Egger	13	-0.001043507	0.00164626	0.539125649	-0.004270176	0.002183162	0.998957037	0.995738928	1.002185546
ieu-b-4834	Liver cell carcinoma || id:ieu-b-4953	Weighted median	13	0.000346529	0.000259117	0.181108965	-0.000161339	0.000854398	1.000346589	0.999838674	1.000854763
ieu-b-4834	Liver cell carcinoma || id:ieu-b-4953	Inverse variance weighted	13	0.000446162	0.000230073	0.052474688	-4.78E-06	0.000897105	1.000446262	0.999995219	1.000897508
ieu-b-4834	Liver cell carcinoma || id:ieu-b-4953	Simple mode	13	0.000204665	0.000477662	0.675897123	-0.000731552	0.001140882	1.000204686	0.999268716	1.001141533
ieu-b-4834	Liver cell carcinoma || id:ieu-b-4953	Weighted mode	13	0.000215491	0.000445877	0.637584107	-0.000658429	0.00108941	1.000215514	0.999341788	1.001090004

## 4 Discussion

This study represents the first comprehensive MR analysis to systematically evaluate the causal relationship between 6 exposure (Alzheimer’s disease, platelet count, ambidextrousness, daily cigarette consumption, alcohol intake, and endocarditis) and the risk of LIHC. Our findings reveal a significant inverse association between genetically predicted platelet count and the likelihood of developing LIHC. This negative correlation is further supported by robust sensitivity analyses. Overall, our MR investigation provides compelling evidence for a unidirectional causal link between platelet count and the risk of LIHC, suggesting that higher platelet count is associated with reduced susceptibility to LIHC.

By focusing on understanding how platelets contribute to carcinogenesis, previous research has suggested that low levels of platelets may increase the risk of developing LIHC (22, 23). Our own study supports this by demonstrating a direct link between reduced platelet count and higher LIHC risk. Nevertheless, it remains uncertain whether decreased platelet count acts as an independent risk factor for LIHC development or simply reflects advanced liver disease which coincides with an elevated incidence of LICH. Contrary to certain observational studies proposing an association between thrombocytosis and greater occurrence of distant metastasis across different types of cancer (24, 25), our current MR investigation does not provide evidence supporting these claims. Additionally, further exploration into the underlying cellular and molecular mechanisms responsible for this phenomenon is necessary. The protective effect of an increased platelet count against LIHC could be mediated through several biological pathways, including but not limited to, the modulation of inflammatory responses and the facilitation of tumor-suppressive immune surveillance. Similarly, the pathways through which alcohol consumption may exacerbate LIHC risk likely involve the induction of hepatic inflammation, oxidative stress, and direct DNA damage.

In our analysis using multiple variables, we discovered a noteworthy link between alcohol consumption and the risk of LIHC in individuals with Alzheimer’s disease. Previous epidemiological studies have consistently reported an inverse correlation between dementia, specifically Alzheimer’s dementia, and the occurrence of cancer (9). A recent meta-analysis also revealed that being diagnosed with cancer resulted in an 11% decrease in subsequent incidence rates of Alzheimer’s disease (26). However, when conducting separate MR analyses on Alzheimer’s disease and alcohol consumption as well as LIHC outcomes, we did not find any significant causal relationship. This lack of significance could potentially be attributed to biases within the study design or limitations associated with studying a European population.

One of the main strengths of this study was the utilization of the MR method to assess the causal relationship between genetically predicted platelet count and the risk of LIHC development within a European population. Moreover, by incorporating multiple samples, we were able to enhance both the overall sample size and precision in estimating causal effects. The implementation of a 2-sample summary MR approach also enabled us to leverage publicly available GWAS data instead of relying solely on individual-level data. In our analysis, summary estimates from individual-level data were obtained; however, researchers can easily apply these methods using the online platform MR-Base.

There are certain limitations that need to be acknowledged in this study. Firstly, the use of summary data restricts the exploration of potential non-linear relationships or stratification effects. Secondly, it is difficult to evaluate the absence of horizontal pleiotropic pathways, and any violation could introduce bias into estimates when using IVW regression. To investigate pleiotropic effects, we employed MR-Egger and weighted median approaches; however, it is important to note that both methods rely on assumptions that cannot currently be tested. Thirdly, due to limited available data for European populations, it is crucial to emphasize that since this study solely focuses on effects within a European population, further justification is required before generalizing these findings to other ethnicities. While our study provides valuable insights into the causal relationships between platelet count, alcohol consumption, Alzheimer’s disease, and LIHC risk among individuals of European descent, the potential effects of population stratification warrant caution. Future research should aim to replicate these findings in diverse populations to ascertain the generalizability of our results.

## 5 Conclusions

In summary, our findings provide compelling evidence supporting a causal relationship between reduced platelet levels and heightened vulnerability to LIHC in the European population. Therefore, it is recommended to prioritize the management of individuals with lower platelet counts to minimize their risk of developing LIHC. As a result, this study contributes to an expanding body of literature indicating that targeting platelet-related agents holds promise as a potential therapeutic approach for early detection and treatment of LIHC.

## Data availability statement

The original contributions presented in the study are included in the article/**Supplementary Material**. Further inquiries can be directed to the corresponding authors.

## Ethics statement

Ethical approval was not required for the study involving humans in accordance with the local legislation and institutional requirements. Written informed consent to participate in this study was not required from the participants or the participants’ legal guardians/next of kin in accordance with the national legislation and the institutional requirements.

## Author contributions

LY: Data curation, Formal analysis, Writing – original draft, Writing – review & editing. LW: Investigation, Methodology, Writing – review & editing. YX: Project administration, Resources, Writing – review & editing. YR: Supervision, Writing – review & editing. TL: Supervision, Writing – original draft. HH: Conceptualization, Funding acquisition, Project administration, Writing – review & editing.
